# Supplementary material for: Pyroptosis of Macrophages Induced by Clostridium perfringens Beta-1 Toxin
Source: Toxins (Basel). 2023 May 29;15(6):366. doi: 10.3390/toxins15060366 (PMC10301120; doi:10.3390/toxins15060366)
Supplement: Supplementary file 1 [file toxins-15-00366-s001.zip › toxins-2374220-supplementary.pdf]

Supplementary Materials:

Table S1: Gene specific primers for qRT-PCR.

Gene	Forward（5′-3′）	Reverse（5′-3′）
GAPDH（human）	TGATTTTGGAGGGATCTCGC	ACGGATTTGGTCGTATTGGG
Gasdermin D	TAGTCCGGAGAGTGGTCCAG	ACCATGAGCTTGAGGGCTTC
IL-18（human）	TGACCAAGGAAATCGGCCTC	ATGGTCCGGGGTGCATTATC
Caspase 1（human）	AATTTTCCGCAAGGTTCGATT	ACTCTTTCAGTGGTGGG
NLRP3（human）	AAAGAGATGAGCCGAAGTGG	TGCACTGGAATCTGCTTCTC
IL-1β（human）	ATGATGGCTTATTACAGTGGC	GTCGGAGATTCGTAGCTGGA
GAPDH（mouse）	GGAGAGTGTTTCCTCGTCCC	ACTGTGCCGTTGAATTTGCC
Gasdermin D（mouse）	GGAGGAATTAATTGAGGCGGC	GGCACCAGTTCTCCAGAGTC
IL-18（mouse）	GGCCGACTTCACTGTACAACC	TCTGGGGTTCACTGGCACTTTG
Caspase 1（mouse）	ACACGTCTTGCCCTCATTATCT	ATAACCTTGGGCTTGTCTTTCA
NLRP3（mouse）	GCTGCGATCAACAGGCGAGA	AAGGCTGTCCTCCTGGCATAC
IL-1β（mouse）	ATAACCTGCTGGTGTGTGAC	TCCTGACCACTGTTGTTTCCC

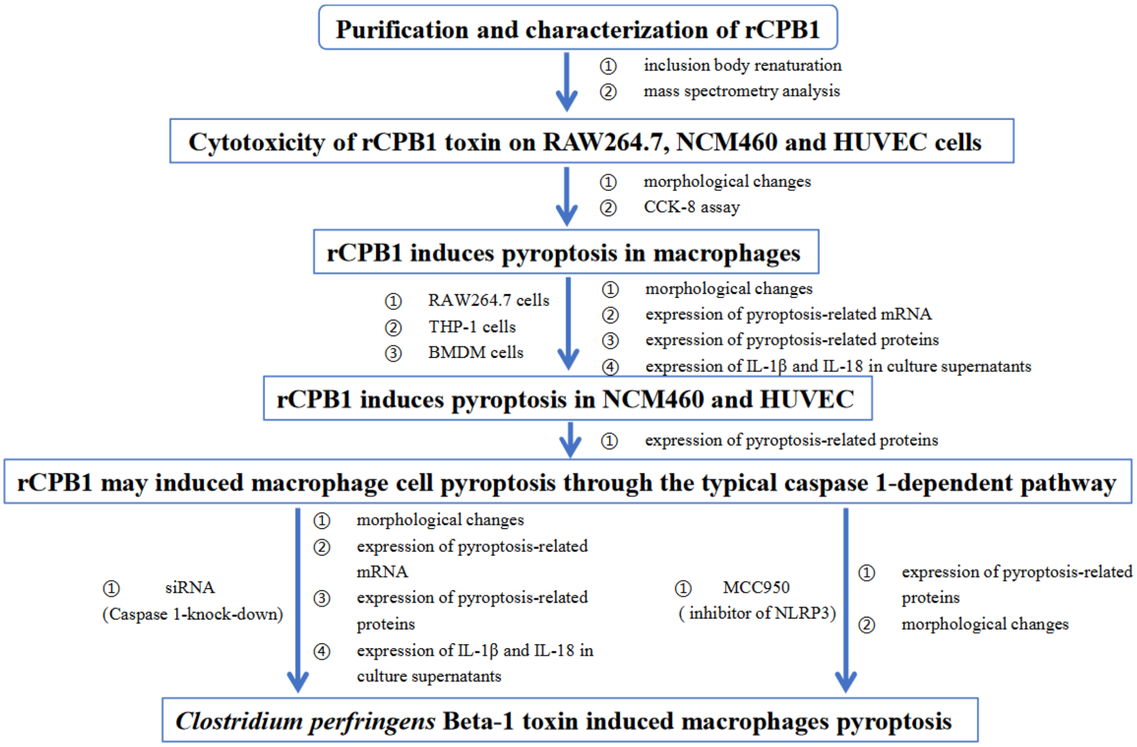

Figure S1: A schematic diagram to summarize our new findings.
